# The Cypriot Indigenous Grapevine Germplasm Is a Multi-Clonal Varietal Mixture

**DOI:** 10.3390/plants9081034

**Published:** 2020-08-14

**Authors:** Apostolis Grigoriou, Georgios Tsaniklidis, Marianna Hagidimitriou, Nikolaos Nikoloudakis

**Affiliations:** 1 Department of Agricultural Sciences, Biotechnology and Food Science, Cyprus University of Technology, Limassol CY-3603, Cyprus; apo_gregoriou@yahoo.gr; 2 Institute of Olive Tree, Subtropical Plants and Viticulture, Hellenic Agricultural Organization ‘Demeter’ (NAGREF), 71003 Heraklio, Greece; tsaniklidis@nagref-her.gr; 3 Department of Biotechnology, Agricultural University of Athens, 10561 Athens, Greece; marianna@aua.gr

**Keywords:** Cyprus, domestication, microsatellites, *Vitis vinifera* subsp. *sativa*, *Vitis vinifera* subsp. *Sylvestris*

## Abstract

Cypriot vineyards are considered as one among the earliest niches of viticulture and a pivotal hub for the domestication and dissemination of grapevine. The millennial presence of *Vitis* spp. in this Eastern Mediterranean island has given rise to a plethora of biotypes that have not been adequately characterized, despite their unique attributes and stress tolerance. This ancient germplasm also has an additional value since it survived the phylloxera outbreak; hence, it possesses a large amount of genetic diversity that has been unnoticed. In order to provide useful insights to the lineage of Cypriot vineyards, a two-year-spanning collection of centennial grapevine cultivars mostly regarded to belong to four indigenous variety clusters (“Mavro”, “Xynisteri”, “Maratheftiko”, and “Veriko”) was initiated. There were 164 accessions across the broader Commandaria wine zone sampled and characterized using a universal microsatellite primer set. Genetic analysis indicated that considered indigenous Cypriot germplasm has a polyclonal structure with a high level of heterozygosity. Moreover, several lineages or unexplored varieties may exist, since a larger than considered number of discrete genotypes was discovered. Furthermore, it was established that grapevine lineages in Cyprus were shaped across eras via clonal, as well as, sexual propagation. The special attributes of the Cypriot landscape are discussed.

## 1. Introduction

Grapes are the most emblematic deciduous woody vines. The imprint of viticulture to agriculture, society, culture, and even religion rituals has been unparalleled, and it can be stated without any reluctance that viticulture is intertwined with human history throughout eras since antiquity. At present, the total global grape production is 75 million tons and the surface of vineyards spans at around 7.5 million ha (FAO). In terms of revenue, the global wine market alone has perceived a stable expansion over the years and has developed as a multi-billion industry [1].

Taxonomically, grapes belong to the Vitaceae family which consists of 14 genera and about 900 species [2]. Based on seed morphological data and fossils, it has been determined that the Vitaceae cluster arose nearly 60 million years ago in the North American region [3]. Nowadays, cultivated species (classified as *Vitis vinifera* L. or *Vitis vinifera* subsp. *vinifera*) have a widespread distribution and can be found across all continents. Nonetheless, *Vitis* spp. progenitors can be exclusively found at specific areas; *V. vinifera* subsp. *sylvestris* (regarded as the Eurasian *Vitis* wild form) is found in North Africa and Eurasia, while *Vitis labrusca* is restricted to Eastern North America. The distinction of wild ancestors from cultivated forms can be challenging, since at least in the broader Mediterranean zone, a wide variety of feral forms and hybrids are present. These comprise cultivation escapees, as well as, seed-disseminated weedy types growing in local niches that occur primarily in surroundings areas of vineyards [4].

Currently, *V. vinifera* subsp. *sylvestris* is considered to be the wild relative of cultivated grapes (*Vitis vinifera* subsp. *vinifera*) and several botanical characteristics are used in order to differentiate the two taxa. One of the most important characters is the flower, as wild grapevines are dioecious, in contrast to virtually all cultivated genotypes that are hermaphrodites [5]. Due to the dioecious manner of the wild grapevine species and the high rates of heterozygosity, asexual propagation was frequently adopted in the grapevine lineage and domestication, in order to preserve desirable traits [6]. Selection of elite traits resulted to a domestication syndrome; thus, generating notable morphological variability in leaves and seeds, as well as a shift from dioecy to self-pollinating hermaphroditism and an increase in berry size and sugar content [7].

Increasing indications converge that the domestication of grapevines was disseminated alongside the advent of wine production from the center of domestication placed at the Near East and the Southern Caspian Belt [8]. The diffusion of grapevines from the principal center of domestication into adjacent areas of Northern Africa and Europe trailed through three main pathways. Firstly, towards Mesopotamia, the broader Eastern Mediterranean Basin and the Southmost Balkans (Pre-Bronze Age). Then, moving via Sicily to Western Europe, and lastly, domesticated grapevine forms reached to Central Europe 1000 years before the common era [9]. Concurrently, introgression of regional forms and secondary domestication events cannot be excluded [10,11,12]. This millennial ongoing process via asexual propagation, as well as trait introgression through inter- and intra-specific crossings resulted in a plethora of discrete and/or overlapping genotypes.

However, there were milestone eras and events that influenced the plethora of genetic grapevine diversity. Early Muslim conquests in the 7th century, and occupation of the primary as well as secondary domestication regions, introduced an extended period of gradual decline of wine production. This in succession could have forced a subsequent extinction of genotypes used for vinification and a turnover towards table grapes production [13]. A more recent substantial reduction of genetic resources in both wild and cultivated grapevines occurred when grape phylloxera outbroken in Europe at the late 1800s [14]. As a result, several American indigenous *Vitis* species were introduced as rootstocks, or used for incorporating disease resistant traits to hybrids [15]. Hence, varieties that were used as scions were mostly preserved but non-grafted genotypes were likely perished. Nowadays, a third wave of genetic erosion occurs via habitat fragmentation of wild and feral *Vitis* forms, supported by an augmented interest of global wine producers for only a handful of elite varieties [16].

Cyprus was on the map of ancient trade routes and, as a result, on the crossroads of the westward dissemination of grapevines to Europe. Cyprus and viticulture have been inextricably related since antiquity [17]. Regardless of its millennial vinification history [18], there is also one paramount reason why Cyprus is a grapevine hotspot of conserved diversity. The phylloxera pest occurs in all Eurasia, but Cyprus is regarded as a protected zone [19]. This constitutes Cyprus as an invaluable genetic resource reservoir of pivotal significance, since the present genotypes are living fossils and epochal remnants of grapevine domestication and distribution. Furthermore, wild grapevines can still be detected in fragmented populations or sporadically across the country; mainly located near small streams or forests. Despite its significance, up till now there has not been any widespread attempt to properly record the diversity of domestic grapevines.

Moreover, a precise number of varieties and clones is not clearly demarcated and there is quite uncertainty regarding the actual number since synonyms and homonyms can occur. Furthermore, ampelographic data are not largely available. Pierre Galet referred that “among the vines of Cyprus, there are (some) fifteen indigenous varieties”: “Xynisteri”, “Mavro”, “Maratheftiko”, “Lefkas”, “Opthalmo”, “Promara”, “Spourtiko”, “Flouriko”, “Yiannoudhi”, “Katomylitiko”, “Kanella”, “Morokanella”, “Michalias”, “Skouro mavro”, “Rodhino rose”, “Rozoudi rose”, and “Maroucho black” [20]. On the other hand, only 12 native cultivars were genotyped by Hvarleva and co-workers [21]. Still, the number of indigenous registered varieties in the national plant variety catalogue is further reduced, probably due to insufficient description.

One of the most emblematic (and globally recognized) dessert wines is Commandaria. Commandaria is an amber-colored traditional wine produced in Cyprus, and it was the first type of wine receiving the controlled appellation of origin certification amid Cypriot wines [22]. One of the aspects that gives Commandaria a unique status comes from the fact that it is one of infrequent cases of wines in production that essentially follow the same principles practiced for millennia. According to Cypriot regulation, Commandaria production is only permitted in an explicit zone of fourteen villages, situated at the slopes of the Troodos Mountain at a spanning elevation from 500 to 900 m.a.s.l. The contemporary vineyard zone covers circa 9000 ha with non-grafted plants. Moreover, Commandaria can be only produced via the vinification of sun-dried grape berries of the two local varieties; Mavro and Xynisteri [23]. Xynisteri is the major white grape Cypriot variety and accounts for 30% of Cypriot vineyards, while Mavro is the prominent black grape variety and occupies almost 40% of Cyprus vineyards. Besides Mavro and Xynisteri varieties that are used in Commandaria, Maratheftiko is also gaining recognition as the most auspicious variety to develop elegant and quality wines. Maratheftiko is a red grapevine variety, and even though it is a cultivated *Vitis* form, it lacks hermaphrodite flowers and is frequently co-planted with other cultivars in order to attain fruit-set and development [24].

Several aspects can affect the outcome of vinification. Among these, primarily the variety (or genotype) and *terroir* are among the most complex and debated issues in viniculture and oenology. The effect of the genotype and the existence of different clones within varieties is often overlooked in studies, hence making it difficult to drive meaningful conclusions (the possible diversity due to genetic background is not considered). Still, clonal selection is extensively practiced in viticulture, signifying that somatic alterations have a substantial unaccounted outcome on berry and wine attributes [25]. Since Cypriot genotypes have been locally cultivated for millennia, it is expected that genetic diversity within variety clusters exist, but largely remain uncharted and unaccredited.

Since a nationwide molecular characterization of the Cypriot germplasm has not been conducted so far, the size of genetic diversity in Cypriot grapevines must be largely underestimated (as indicated by the low and uncertain number of varieties reported, compared to other countries having similar size and geography). Nowadays newly established vineyards are propagated clones of the same genotype; hence, focusing on antique grapevines could in fact be more effective in demarking genetic diversity. The hypothesis when commencing this project was that relic genotypes that are currently considered as a variety (due to the lack of robust ampelographic description and identification) could in fact be landraces (populations), discrete clones, or different varieties that may have a potential in enriching the Cypriot grapevine germplasm. Towards attaining meaningful insights for the Cypriot grapevine landscape, 164 centennial genotypes (putatively belonging to four local Cypriot varieties; Mavro, Xynisteri, Maratheftiko, and Veriko) were sampled across the Commandaria zone and genotyped using 11 nuclear SSRs in order to reveal their genetic variability. This study is the starting point towards the prominence and safeguarding of local indigenous germplasm, and the highlighting of historical knowledge and heritage concerning the millennia viniculture of Cyprus.

## 2. Materials and Methods

### 2.1. Plant Material

**Figure 1:**
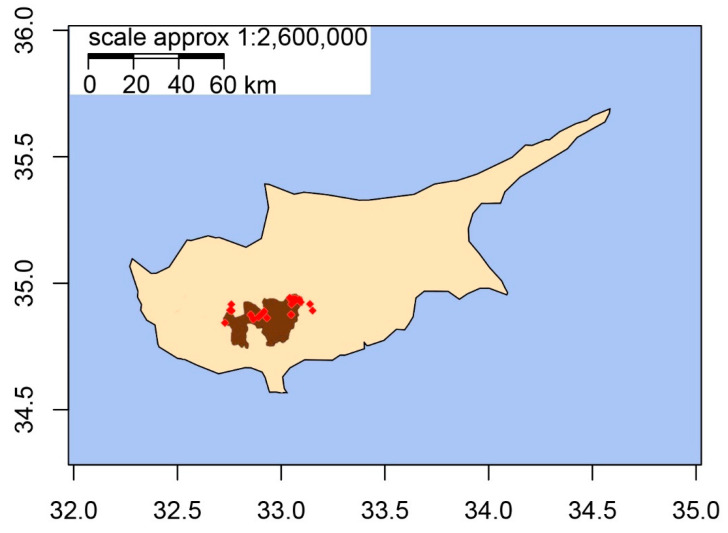
Map of Cyprus. Shaded area (brown) indicates the Commandaria zone and red rhombi mark the collection sites.

A total of 164 centennial grapevine accessions putatively attributed to the four mentioned populations, Maratheftiko, Mavro, Veriko, and Xynisteri (Table S1), were collected across Cypriot vineyards (Figure 1). Sampling was conducted across the seven wine-zone districts in the Commandaria region and the wine villages of Cyprus, during a spanning period of two years (2018–2019). Vines were carefully chosen through the support of local industry members and vineyard owners to certify a record of vine age, while distinctive phenotypic characters were considered according to the growth stage (Supplementary Data).

### 2.2. DNA Extraction Protocol

For DNA extraction, leaves were excised and kept between humid paper towels on ice, until storage at −80 °C. Leaf tissue (100–200 mg) was weighed in 2-mL rounded Eppendorf tubes, flash frozen in liquid nitrogen and freeze-dried overnight. Two stainless steel balls (3 mm) were added in each Eppendorf, samples were quickly frozen in liquid nitrogen, and tissues were crushed for 30 sec (at full speed) using a mixer mill MM 200 (Retsch). Samples were kept at −80°C until DNA extraction. In preliminary tests, it was established that DNA extraction using standard DNA extraction procedures (CTAB or commercial DNA extraction kits) proved problematic (for multiplex PCR) due to the highly recalcitrant nature of vine tissues. Hence, before DNA extraction, crushed tissues were pre-washed twice with sorbitol wash buffer (Sorbitol wash buffer: 100 mM Tris-HCl pH 8.0, 0.35 M Sorbitol, 5 mM EDTA pH 8, 0.1% (w/v) Polyvinylpyrrolidone PVP-40, 0.1% β-mercaptoethanol) to remove interfering metabolites [26]. After the wash of the inhibitors, cell lysis was achieved using a high-salt CTAB extraction buffer (2% CTAB (hexadecyltrimethylammonium bromide), 100 mM TrisHCl pH = 8, 20 mM EDTA, 1.4 M NaCl, 0.1% (w/v) Polyvinylpyrrolidone PVP-40, 0.1% β-mercaptoethanol). Impurities were further removed using equal volumes of chloroform: Isoamyl alcohol (24:1) and DNA were precipitated with saturated NaCl (1/10th of the lysis buffer volume) and ice-cold ethanol (twice of the lysis buffer volume). The pellet was washed twice with 100 ul of 70% cold ethanol, air dried, and dissolved in 100 μL of dd H_2_O. DNA concentration and purity were estimated using nanodrop spectrophotometry.

### 2.3. Microsatellite Genotyping

Eleven SSR markers were selected for the analysis of the Cypriot germplasm (Table S2). Nine of them are suggested within the European projects Genres081 and GrapeGen06, proposing the use of a common set of microsatellite markers [27]. All forward primers employed were designed with a M13(-21) tail at the 5′-end and carried a fluorescent dye label (FAM^TM^, ROX^TM^, or TAMRA^TM^). This enabled the run of a one-tube, single-reaction nested PCR, as previously described [28]. Based on expected allele sizes from databases (Italian Vitis Database, http://www.vitisdb.it/; Vitis International Variety Catalogue VIVC, http://www.vivc.de/) and pilot single locus PCR reactions, three different panels were selected (Table S2), in order to ensure non-overlapping allele sizes across loci. Furthermore, even though green fluorescent dye (JOE^TM^) was initially tested for multiplexing, it was not finally employed, since it was determined that it interfered with red (ROX^TM^) and blue (FAM^TM^) spectra, causing minor peak pull-ups that could result to erroneous scoring.

For PCR reactions, the mix contained 50 ng of template DNA, 10 pmol of a labeled M13 tailed forward primer, 10 pmol of the reverse and 2.5 pmol of the forward primer, 0.2 mM dNTPs, 0.5 U KAPA Taq DNA Polymerase (Kapa Biosystems), and a 2.5 mM final concentration of MgCl_2_ in a 12 μL final reaction volume. Conditions for the PCR amplification were: 94 °C (5 min for initial denaturation), followed by 39 cycles at 94/56/72 °C (60 s), and a final extension at 72 °C for 30 min.

Amplification products were verified using a standard 2% agarose electrophoresis and diluted at a 1:40 ratio with dd H_2_O. One μL of the dilutions was added to 10 μL deionized formamide and 0.2 μL of DNA size standard (GeneScan 500-LIZ, Applied Biosystems, Foster City, CA, USA), before denaturing at 95 °C (5 min). Allele fragments were separated (in three discrete panels) by capillary electrophoresis using an Applied Biosystems 3130^®^ Genetic Analyzer (Applied Biosystems, Foster City, CA, USA). Two control varieties (Cabernet Sauvignon and Merlot) and four Cypriot genotypes were replicated in every run, to normalize microsatellite allele sizes and associate genotypes across runs and databases. Allele scoring was performed by two researchers independently, and tandem software was utilized to verify/correct bins [29].

### 2.4. Genetic Relationships and Analysis of Population Structure

Microsatellite data curation and formatting was performed via the MS Excel add-in GENALEX v. 6.501 [30]. All genotypes were included for calculating allelic frequencies across loci studied. In order to assess the discriminating power among unique genetic profiles, a genotype accumulation curve was constructed. Additionally, the number of multi-locus genotypes (MLGs), as well as the number of expected MLG (eMLGs) were calculated. Moreover, genotypic diversity was assessed with several indexes (H: Shannon–Wiener Index of MLG diversity, G: Stoddart and Taylor’s index of MLG diversity, lambda Simpson’s index, E.5: Evenness of the alleles, and Hexp: Nei’s unbiased gene diversity).

The same dataset was similarly used to test for linkage disequilibrium and Hardy–Weinberg equilibrium (HWE) in the grapevine accessions. Genetic relationships between individuals (MLGs) were assessed using the dissimilarity distance calculation and visualized as a minimum spanning network (MSN) and a discriminant analysis of principal components (DAPC). All of the above-mentioned statistics/analyses were performed using the Poppr (V. 2.8.5) package [31] and the RStudio suite (V 1.2.5033; R V 3.6.2).

A phylogenetic tree was also constructed using the binary template (converted from allele size) using the R package polysat [32]. An approximate likelihood-ratio test (aLRT) for branch support was achieved by means of the SH-like parameter as previously described [33]. The newick -formatted tree was displayed and manipulated using the iTOL v4 server [34].

Finally, a Bayesian statistic employing method for estimating genetic kinship was performed using Structure 2.3.4 [35]. The admixture model was selected and 20 independent repeats per K value (extending from 1 to 20) were run. Each run involved 100,000 iterations burning period and a post burning simulation of 1,000,000. Validation of the most probable number of clusters K and visualization was achieved using the Clumpak server (http://clumpak.tau.ac.il/).

## 3. Results

### 3.1. Genetic Affiliations across the Cypriot Germplasm

From the broader Commandaria zone of Cyprus 164 vines putatively attributed to four varieties (Maratheftiko, Mavro, Veriko, and Xynisteri) were genotyped using a primer set of 11 SSR markers. The genotype accumulation curve (Figure S1) indicated that the 11 microsatellite loci were acceptable in order to delineate all the multi-locus genotypes (MLGs) present among cultivars (Table 1). The probability of identity (PI) of two samples to have the same genotype was also calculated for the dataset, and it was concluded that the cumulative capacity of the 11 loci resulted to a PI value of 6.2 × 10^−7^ to 7.8 × 10^−10^ across populations.

All loci were found polymorphic (Table S3), and in total 102 alleles were detected, varying from six at loci VrZAG112 and VVMD25 up to 16 at locus VVMD28; with a mean of 9.27 alleles per locus (Table 2). Even though many discrete alleles were detected across loci, a few alleles were predominant, while the rest were noticed at lower frequencies (Table S4). After clone correction filtering (removal of redundant genotypes), several diversity indices were established across loci (Table 2).

The Simpson index varied from 0.5 for locus VrZAG112, up to 0.83 for locus VVMD28 (mean 0.73); in general, being proportional to the number of alleles detected among discrete loci. Still, across loci the high percentages of heterozygosity and evenness of alleles, elevated the efficiency of these markers to reveal the genetic polymorphism. Indices indicated the high potential for this marker set to define variability in the Cypriot panel of grapevines.

Multi-locus genotype analysis across populations recorded 32 MLGs for Xynisteri, 41 for Mavro, eight for Maratheftiko, and two for Veriko (Table 1). The most frequent MLG across populations was MLG 76 (16 vines) and MLG 81 (14 vines) that were detected for Xynisteri (Table S3). Correspondingly, several accessions were also found to be common in Mavro, (MLG 38 in 14 vines) and Maratheftiko population (MLG 8 in 7 vines), but at a lesser extent.

Even though genotypic abundancy can be primarily inferred from the number of MLGs detected, unequal sample size can cause a partial bias; hence, an estimate of the quantity of genotypes that could be anticipated at the major common sample size established on rarefaction (eMLG) could be more suitable. The expected number of eMLGs was also computed and was found comparable among Xynisteri and Mavro populations (10; Table 1). Still, a somewhat higher amount of genetic diversity was established in the Mavro cluster (*H* = 3.714, *Hexp* = 0.667), even though many accessions shared a genotype.

In order to determine the mode of reproduction across these centennial varieties, a test of linkage disequilibrium was conducted for clone corrected genotypes. Consequently, disequilibrium indices were calculated (*Ia* and *rbarD*); it seems that populations in Cyprus were mostly clonally propagated. Moreover, we explored the probability that loci were under Hardy–Weinberg equilibrium (Table 1). Ιt was established that several loci were in HWE (*p* < 0.5). This suggests that even though at a reduced rate, sexual propagation has also occurred in the lineage of Cypriot grapevine varieties.

### 3.2. Population Structure of the Main Cypriot Grapevine Varieties

**Figure 2:**
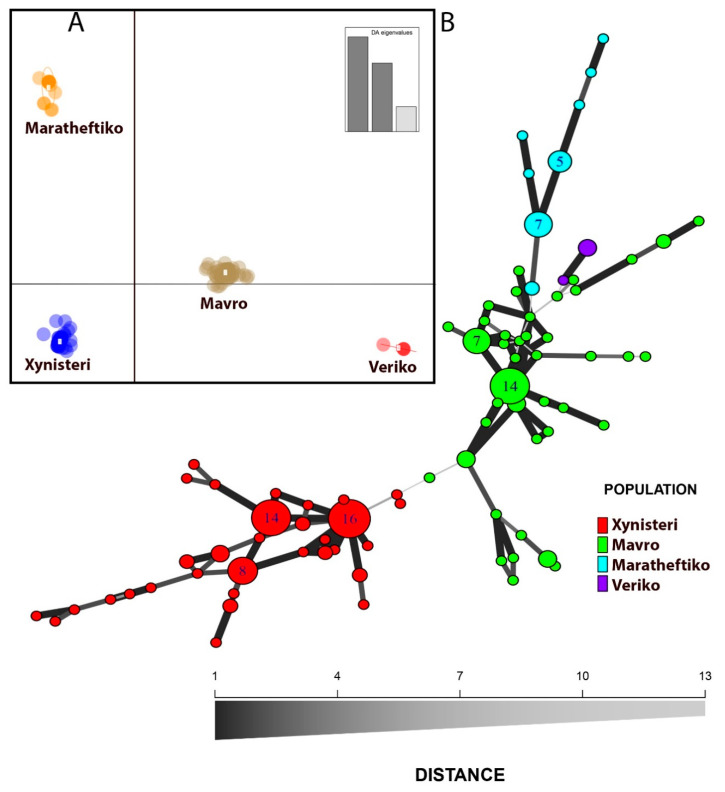
(**A**) Discriminant analysis of principal components (DAPC) depicting a clear cut-off across populations (DAPC cross-validation values indicated that the proportion of successful prediction (larger than 0.8) was optimum for 10 PCA axes). (**B**) Minimum spanning network (MSN) of the Cypriot grapevine varieties studied. Linear and reticulated affiliations are evident across Xynisteri (red), Mavro (green), Veriko (blue), and Maratheftiko (purple) clusters. Nodal size is proportional to the number of accessions sharing an MLG.

Estimates of population differentiation across loci (Figure S2) were elevated suggesting that a great amount of genetic diversity still exists within each considered cluster. In order to infer the number of groups of genetically related accessions, a multivariate approach partitioning between- and within-group components was used. The discriminant analysis of principal components (DAPC; Figure 2A) revealed that a clear genetic distinction across the Cypriot populations studied is evident. Moreover, it was established that significant within-variability also exists. The minimum spanning network (MSN) analysis (Figure 2B) depicted a structure having limited reticulation, indicative of clonal propagation where somatic mutations can result in novel genotypes that are highly affiliated to the core. Still, at low levels, reticulation was evident among genotypes. In general, the four main clusters of genotypes distinguished reflected the discrete genetic background of the four Cypriot strains studied. Interestingly, linear and reticulate relations were depicted within the germplasm of Xynisteri and Mavro populations, while the Maratheftiko cluster had a lower kinship to the core of genotypes.

**Figure 3:**
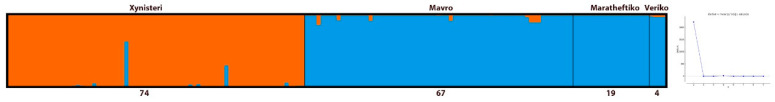
Structure analysis for Cypriot grapevine germplasm at K = 2 cluster and delta K probability for Cypriot genotypes. Numbers correspond to the analyzed samples.

The population structure among the Cypriot collection was also assessed using the Bayesian procedure STRUCTURE (Figure 3). The optimal for the ad hoc number, based on the second order rate of probability of the likelihood function respecting to Delta K, was attained for K = 2. At low cluster complexities, this analysis, indicated two discrete genetic groups (Delta K = 2229.2). The majority of putatively Mavro, Maratheftiko (black berry varieties), and Veriko (rose berry varieties) accessions were found highly constrained (all having a mean proportion of membership higher than 0.9, and were clustered in the first genetic category (Table S5). On the other hand, genotypes regarded as Xynisteri (white berry variety) had lower affinity to the first group and were affiliated with the second genetic ancestry. Within each population there were instances of admixture genotypes, characteristic of sexual propagation. As the level of K complexity increased (higher K clusters), a finer and a more detailed structure was portrayed (also differentiating Veriko genotypes; data not shown).

Finally, a phylogenetic tree was constructed in order to also visualize the genetic relationships across genotypes using a hierarchical clustering approach with branch support (Figure S3). The dendrogram produced was analogous to the previous analyses, though with a notable distinction. The Maratheftiko cluster was not positioned as an outgroup as in Figure 2, but had a greater affinity to Mavro group, as it was placed among Mavro accessions. Moreover, Veriko accessions were highly affiliated to Mavro genotypes. Still, there were a few instances where several genotypes were sporadically clustered to different than expected groups, showing that misnaming is possible, or that these genotypes could in fact be different than registered varieties.

## 4. Discussion

The main scope of the current study was to evaluate the level of existing genetic diversity across Cypriot vineyards in the broader Commandaria area, and possibly identify novel genotypes that could provide evolutionary insights of the Cypriot grapevine germplasm structure. It was also intended to delineate the extent and imprint of genotype variability in one of the world’s oldest niches of viticulture and pivotal landmark of grapevine domestication. Despite the fact that Cyprus is proximate to the grapevine center of domestication and a vital region of *Vitis* sp. westward dissemination, a very low number of native varieties has been reported so far [20,21]. Furthermore, it is speculated that the lack of robust ampelographic descriptors permits the unnoticed misuse of several “alike” forms as the same variety. As a result, there is the possibility that grapevine variability in the form of landraces, clones, or novel varieties remains largely unnoticed till today.

Towards that objective, two-year genetic resources collecting across the vineyards of Cyprus in the broader Commandaria zone was initiated and 164 centennial grapevines were obtained. The samples were genetically characterized using a universal set of microsatellite primers at eleven loci. Genotypic fingerprinting using microsatellite markers can deliver a valuable tool that allows for inter-accessions assessment of discrete collections, as well as, the pedigree analysis of hybrids and cultivar certification [36,37,38].

Among the 164 Cypriot accessions (registered and putatively assigned to the four clusters), a total of 83 multi-locus genotypes (MLGs) were identified (Table 1). Interestingly, 64 MLGs were unique and thus were detected only in one individual. As a consequence, 19 MLGs were found to be common (at different frequencies) among the remaining accessions. Hence, it was established that centennial grapevines of Cyprus are characterized by extensive genetic diversity and a small fraction of the sampled grapevine collection was composed of redundant germplasm. In a recent study, Drori and co-workers [13] collected and characterized grapevine genetic resources in Israel (a proximate to Cyprus region, having similar size and edaphoclimatic conditions). It was also concluded that a large proportion of the *V. vinifera* subsp. *sativa* and *V. vinifera* subsp. *sylvestris* genotypes had a unique genetic profile (about 40%) and did not correspond to known varieties/genotypes.

In viticulture, it is known that grapevine cultivars frequently consist of discrete clones, sharing mutual phenotypic traits and grouped as a variety cluster [39]. If, however, clones belonging to a cluster have traits discrete enough they are considered as different varieties. Nevertheless, several genetically affiliated varieties are very similar morphologically and hence hard to distinguish based on visual observation [40]. Conversely, clones of varieties can considerably differ in several characteristics without a significant change in genetic profiles [41]. Microsatellites can be proven a valuable tool delineating the above-mentioned dilemma, since such markers are extremely informative for attaining the level of heterozygosity across and within grapevine varieties. Across grapevine genotypes, a mean of nine alleles per locus [42] and a maximum of 23 alleles has been reported [41]. In the present study, 11 microsatellite loci were used, and a comparable level of allelic diversity was attained. An average of 9.27 alleles was obtained while in the case of the most polymorphic marker (VVMD28), 16 discrete alleles were recorded (Table 2). Moreover, several genotypes that were putatively assigned to the four clusters had extensive allelic discrepancies; hence, the possibility they are misidentified as the aforementioned cultivars and are in fact discrete varieties cannot be uncritically ruled out.

In genetic studies, when dealing with clonal taxa, analyses can be typically conducted using (or not) clonal correction. Since allele frequencies can be affected by the number of redundant genotypes, clone correction is often advised in order to better depict genetic relationships and kinships, as well as removing potential bias [31]. In the current survey, clone correction was performed in order to robustly estimate genetic indices (Table 1 and Table 2). A substantial level of heterozygosity was revealed within the Cypriot varieties (*Hexp* = 0.732). The within cultivar heterozygosity is infrequently reported across studies, but in several cases, it seems that a moderate-to-high proportion of heterozygosity exists. Heterozygosity levels across grapevine clones generally vary from 0.47 for “Tannat” [43] to 0.87 for “Pinot Noir” and “Riesling” [44] cultivars, having a mean of 0.77 [40]. Despite the fact that data across studies are somewhat difficult to compare, since there are discrepancies in the number of loci and accessions employed, several common conclusions can still be attained [16]. In general, even though reports employ grapevine datasets of a few dozens to more than thousand accessions (focusing on eight to eleven loci), average to elevated heterozygosity values are reported [42,45,46,47,48,49]. Thus, it seems that diversity is present within cultivars, as well as across cultivars.

Since Cypriot grapevines have been cultivated for millennia, a high amount of diversity is expected among clones or varieties. Pinot is another characteristic cultivar where several clones are considered, and a great amount of heterogeneity exists. Unfortunately, the lack of precise ampelographic data on Cypriot germplasm complicates the cut-off line that defines the optimal taxonomic status. As a result, the existence of unidentified/misidentified varieties, hybrids, or feral forms considered as a “true-to-type” variety is a possibility. In the current study the predominant MLGs of Xynisteri cluster (MLG 76, MLG 81, and MLG 79) differed in one out of 11 loci, hence probably representing different clones of the same variety. This was also the case for the Mavro (MLG 38 and MLG 36) and Maratheftiko (MLG 7 and MLG 8) clusters, which were also identical in 10 out of 11 loci. Conversely, in the study of Hvarleva et al. [21], genotypes referring to discrete varieties differed at almost half of the analyzed loci in bilateral comparisons. In an equivalent study utilizing clones of Pinot, which is generally considered as one of the oldest varieties, significant discrepancies of SSR fragment lengths within clones of “Pinot gris” (loci VVS2, VMCNG1E1, VMC8A7, VMC7G3, VrZAG79, and VrZAG 25) and Pinot noir (loci VVS2, VVIM10, VMCNG1E1, VMC1F10, VMC2H4, VMV8A7, VMC7G3, VVMD28, and VrZAG 25) were established [50]. Still, in the case of Cypriot germplasm, a clear demarcation of what constitutes a variety, clone, or a landrace (population) is not an easy task, especially due to the lack of robust ampelographic data. This can be depicted in the DAPC (Figure 2A) that clearly demarks four clusters. Still, the within-group variation seems relatively small and a confident conclusion of the cut-off line separating clones from varieties cannot be established. In fact, one variety may consist of a smaller or larger number of similar and more or less related clones. Nevertheless, the findings presented here showcase beyond any doubt that a much larger than currently considered genetic variation is present across Cypriot vineyards and that several grapevines varieties are in fact misidentified by farmers.

The high amount of heterozygosity within Cypriot-cultivated grapevines could also be the result of a recurrent hybridization and parallel domestication events due to the long cultivation history and the proximity to the hotspot of grapevine domestication. In that direction, in our dataset several tri-allelic profiles across markers and genotypes were observed for markers VVMD32 and VVMD5, indicative of hybridization or chimerism. This phenomenon in clonally propagated grapevines such as monumental cultivars could participate in clonal variation and hamper proper variety identification or pedigree analysis [51]. Zarouri and co-workers [52] have also reported that amplification of multiple alleles per locus in one accession is possible. Feral forms and wild grapevines are still present in Cyprus, and sporadically, individual plants have been marked in remote regions near riverbeds.

Unfortunately, microsatellite profiles of Cypriot grapevine genotypes (using a universal primer set) are not available in public databases such as VIVC. Furthermore, partial comparison of the current dataset to the one previously described [21], revealed discrepancies in allele sizes or shifts. Such disparities are often reported in microsatellite markers studies, since inter-laboratory protocols can influence the outcome; the use of different DNA polymerase types, PCR elongation time, template concentration, or different fluorochromes can affect the addition of an adenine nucleotide at the 3′ end, resulting in allele size shifts due to the differences in molecular weight or even spectra pull-ups [53]. Hence, comparison across inter-laboratory studies must be cautiously regarded. Additionally, screening of the profiles attained in the current study to the VIVC database did not reveal similarities. The novelty of these centennial genotypes is further supported by the fact that foreign cultivars were only fairly recently (1970s) introduced into Cyprus [21].

Nonetheless, the genetic relationships of the current dataset largely correlates to previous studies that included Cypriot genotypes [21] even though a different primer set was used. Mavro and Maratheftiko populations seem to be genetically affiliated compared to Xynisteri that was the most genetically distant form, from the core cluster of Cypriot genotypes. Maratheftiko, or locally known as “Vamvakada” (due to a white coating at the back of its leaves resembling cotton; vamvaki is the Greek word for cotton) is an irregular occurrence of grape variety. Maratheftiko is one of the rare cases of grapevine cultivated forms that lacks hermaphrodite flowers. More specifically, its flowers present a well-developed pistil, but the stamens are reflexed; hence, Maratheftiko is incapable of self-pollination. This is the main problem hampering its cultivation in Cyprus despite being a drought tolerant variety and having exceptional vinification capabilities. Maratheftiko is considered among the most promising Cypriot varieties across local wineries since it produces red wines with an intense full body, having soft tannins and unique aromas when harvested and vinified correctly [54]. In the current survey it was clustered as an outgroup, having a limited genetic affiliation to the core of other Cypriot genotypes (Figure 2B). The Hardy–Weinberg equilibrium (HWE) indicates that at least in the case of the Maratheftiko cluster, widespread hybridizations have occurred throughout its lineage. In that direction, previous studies employing SNP markers have described a Levant domestication of the vinifera cluster and have displayed signs of introgression from local *sylvestris* forms as grapevines disseminated to Europe [12]. The extent to which local wild forms contributed to the establishment of the grapevine germplasm globally remains a disputable subject [9].

Still, the indices of association and significant *rbarD* values indicated that Cypriot genotypes were also clonally propagated since a considerable disequilibrium among loci was attained signifying conscious selection of elite genotypes during domestication. The clonal propagation of Cypriot genotypes can also be depicted from the network analysis since a limited reticulation can be observed among clusters (Figure 2), even though at slower rates compared to sexual propagation, clonal propagation can give rise to genetic variation [55]. Clonal polymorphism within perennial species has been mainly attributed to naturally occurring mutations throughout grapevine growth [39].

The “wild” characteristics of the Cypriot germplasm is also supported by vinification features of these genotypes. Xynisteri (‘Xyno’ is the Greek word for sour) is the predominant white variety and has a sharp taste due to the high level of acids [54]. Other traits indicating that Xynisteri retains some wild type features is the exceptional resilience against drought and elevated temperature [56], which are frequent features across wild *V. vinifera* spp. *sylvestris* forms [57]. In fact, even though domestication has resulted into higher yield, larger berries, and a higher sugar content, this selection was at the expense of biotic and abiotic stress capacity [9]. Recent studies have stressed the significance of wild forms for breeding purposes against mildew pathogens [58,59] or abiotic resilience and berry quality [60]. Past pedigree records and passport data (http://www.vivc.de/) reveal that Xynisteri has been broadly used in hybrid crosses in Israel during the 1930s, establishing a series of Hebron varieties used mainly as rootstocks. Nowadays, Xynisteri is largely tested outside the narrow range of Cyprus, at high temperate-arid regions of Australia, highlighting the ready-to-use potential of Cypriot germplasm for grapevine sustainability purposes [56].

In terms of conservation, several projects for the preservation and highlighting of local genetic resources have been directed in grapevine growing countries [16]. As a result, a significant number of minor varieties have been collected and characterized. Since several elite foreign varieties were only fairly recently (1970s) introduced into Cyprus [21], the Cypriot germplasm largely preserves its original structure throughout antiquity and is a remnant of the westward grapevine domestication and dissemination. In conclusion, the genetic characterization of such genetic resources is of paramount importance not only having a local interest but extendable to global viticulture. Furthermore, in the current study it was portrayed that the existence of a plethora of discrete genotypes is in fact an indication that more Cypriot varieties can exist and the need for proper ampelographic characterization is imperative.

## Figures and Tables

**Table 1:** Genetic variability across Cypriot grapevine populations. Total number of discrete genotypes and genetic diversity indices (clone corrected).

	Pop	*MLG (N)*	*eMLG*	*H*	*Hexp*	*Ia* *	*rbarD* *	*Ho*	*He*
1	Xynisteri	32 (74)	10	3.466	0.489	1.03	0.118	0.700	0.430
2	Mavro	41 (67)	10	3.714	0.667	4.141	0.427	0.923	0.626
3	Maratheftiko	8 (19)	8	2.079	0.567	−0.395	−0.148	0.981	0.526
4	Veriko	2 (4)	2	0.693	0.652	NA	NA	0.932	0.474
5	Total	83 (164)	10	4.419	0.732	4.143	0.42	0.884	0.514

*MLG* = number of observed multi-locus genotypes, *N* = total number of samples, *eMLG* = expected multi-locus genotype, *H* = Shannon–Wiener index of MLG diversity, *Hexp* = Nei’s 1978 expected heterozygosity, *Ia* = the index of association, *rbarD* = the standardized index of association * *p* < 0.001, *Ho =* observed heterozygosity (calculated by GeneAlEx), *He* = expected heterozygosity (calculated by GeneAlEx).

**Table 2:** Allele summary statistics for the 11 analyzed loci of Cypriot grapevine populations.

*Locus*	*Alleles*	*1-D*	*Hexp*	*Evenness*
VVMD27	7	0.75	0.75	0.8
VrZAG79	13	0.82	0.82	0.7
VrZAG67	12	0.71	0.71	0.64
VrZAG62	8	0.81	0.81	0.89
VrZAG112	6	0.5	0.5	0.52
VVS2	7	0.68	0.68	0.68
VVMD5	8	0.79	0.8	0.86
VVMD7	8	0.75	0.75	0.75
VVMD28	16	0.83	0.83	0.68
VVMD25	6	0.58	0.58	0.65
VVMD32	11	0.8	0.8	0.8
mean	9.27	0.73	0.73	0.73

*Alleles* = number of observed alleles, *1-D* = Simpson index, *Hexp* = Nei’s 1978 gene diversity, *Evenness* = distribution of genotype abundances.

## Supplementary Materials

All relevant data are appended as supporting information. The following are available online at https://www.mdpi.com/2223-7747/9/8/1034/s1. Supplementary data: Morphological characters used for the discrimination of varieties/ Leaf diversity across accessions. Figure S1: Genotype accumulation curve depicting the efficiency of SSRs in delineating the Cypriot Vitis spp. Genotypes. Figure S2: Genetic variability estimates from clone corrected genotypes. Figure S3: Phylogenetic tree of Cypriot grapevine genotypes. Table S1: Sampling locations and germplasm information across collection sites within the Commandaria zone. Table S2: Primers and panels used for the amplification of alleles across 11 loci. Table S3: Alleles size and MLGs across the 164 Cypriot genotypes sampled and analyzed. Table S4: Frequency of alleles detected for every locus in the Cypriot grapevine collection. Table S5: Ancestry probability of STRUCTURE analysis across the 164 Cypriot genotypes.
