# Supplementary material for: The Cypriot Indigenous Grapevine Germplasm Is a Multi-Clonal Varietal Mixture
Source: Plants (Basel). 2020 Aug 14;9(8):1034. doi: 10.3390/plants9081034 (PMC7463456; doi:10.3390/plants9081034)
Supplement: Supplementary file 1 [file plants-09-01034-s001.zip › plants-821124 - supplementary figures for XML.pdf]

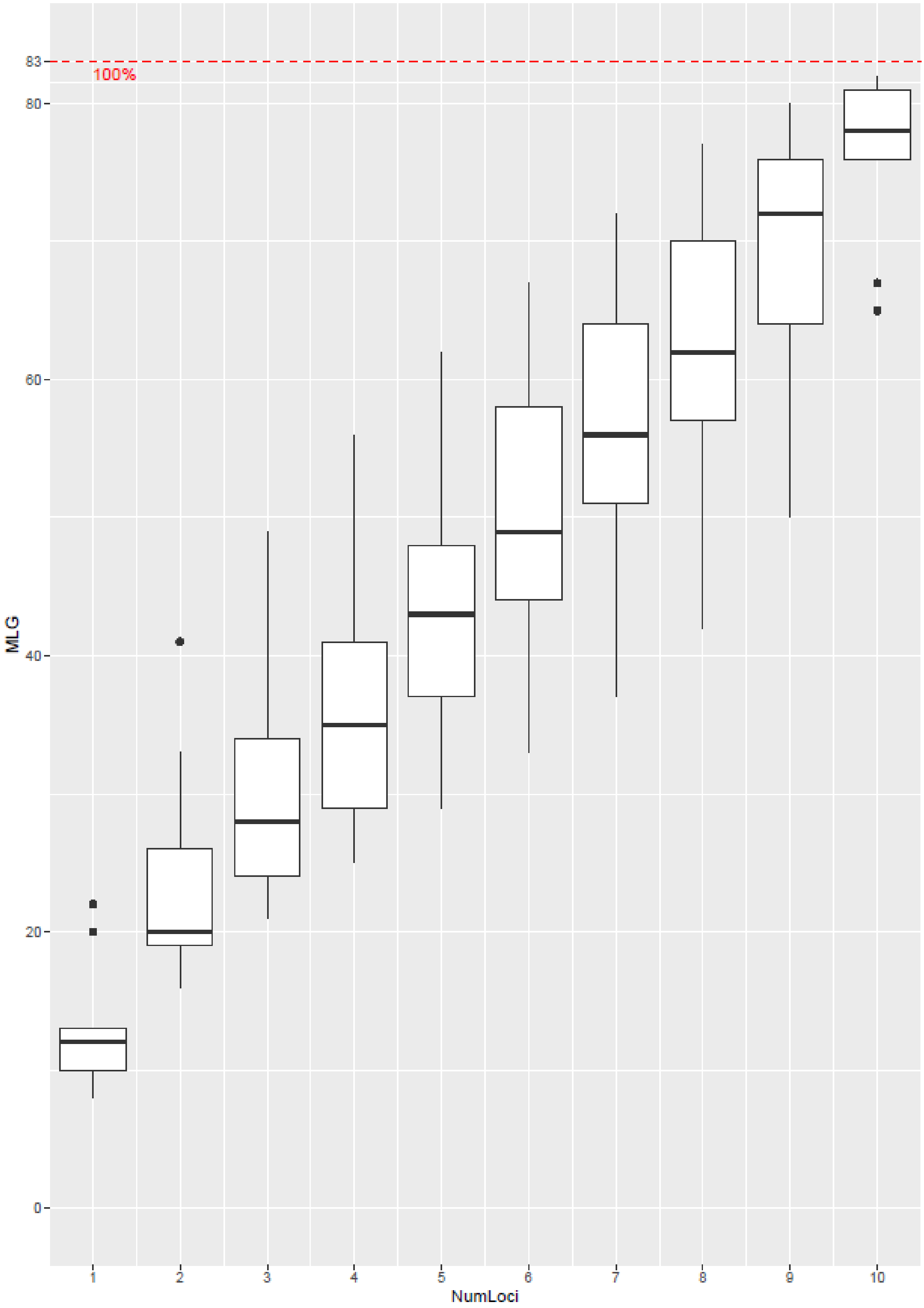

Figure S1. Genotype accumulation curve depicting the efficiency of SSRs in delineating the Cypriot *Vitis* spp. Genotypes.

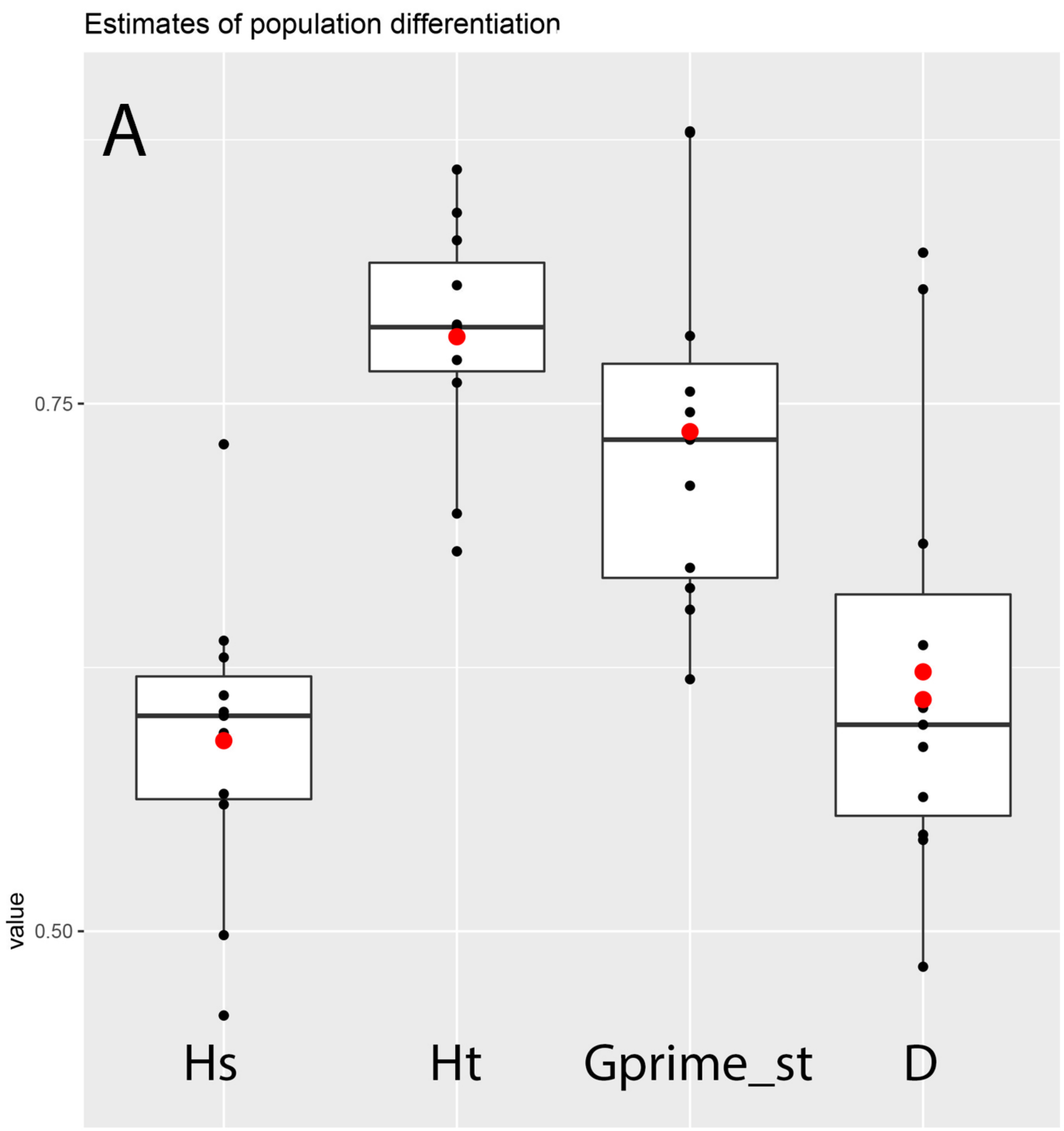

Figure S2. Genetic variability estimates from clone corrected genotypes. Boxplots depict the distribution of genetic indexes across the 11 loci studied. Hs: Heterozygosity with population structure, Ht: Heterozygosity without population structure, Gprime_st: Hedrick index, D: Jost index (all indexes were calculated using the Poppr package).

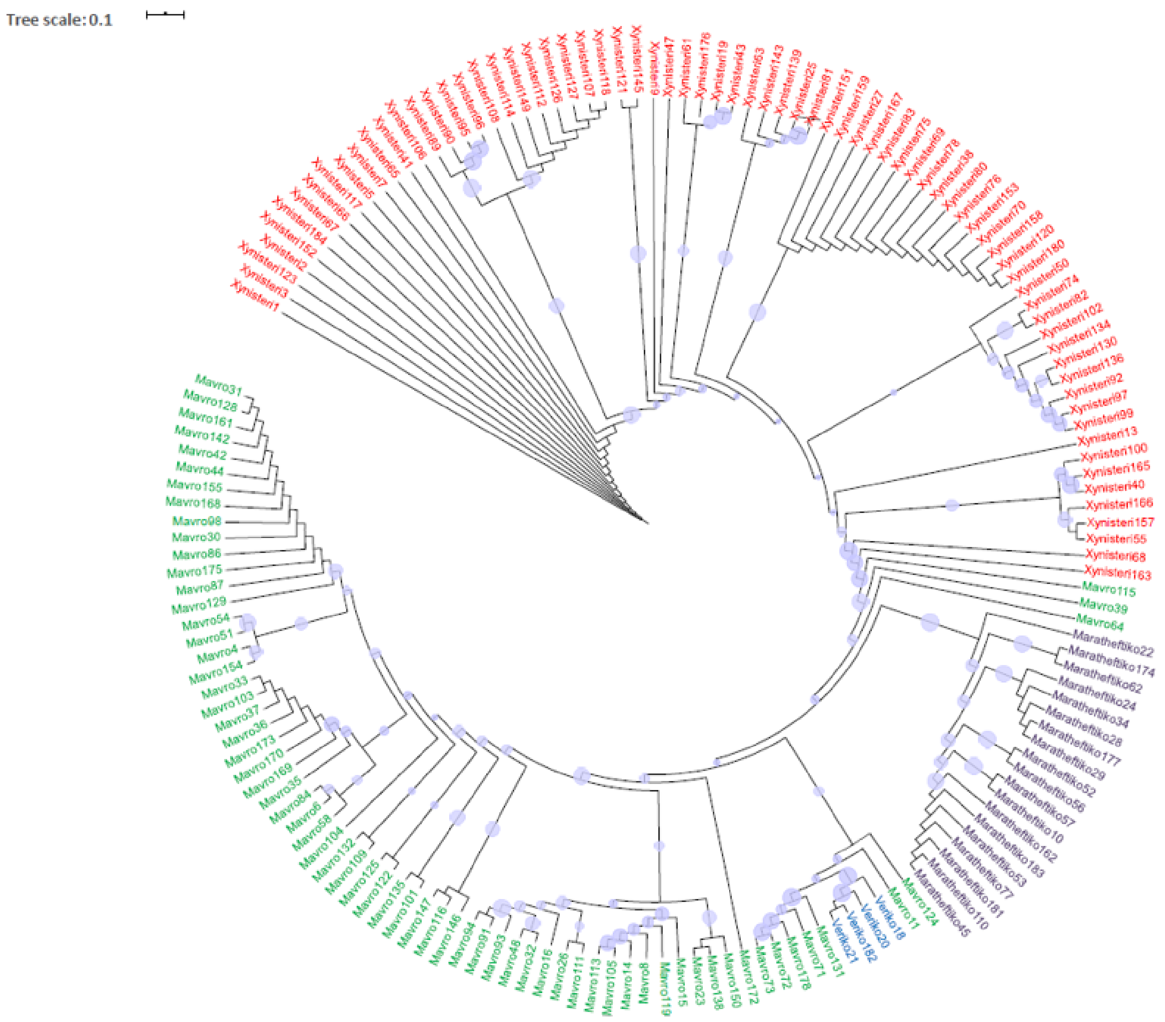

Figure S3. Phylogenetic tree of Cypriot grapevine genotypes. The size of nodes correlates to bootstrap support values.
