# Supplementary material for: The Cypriot Indigenous Grapevine Germplasm Is a Multi-Clonal Varietal Mixture
Source: Plants (Basel). 2020 Aug 14;9(8):1034. doi: 10.3390/plants9081034 (PMC7463456; doi:10.3390/plants9081034)

Morphological characters used for the discrimination of varieties.

	Xynisteri	Mavro	Maratheftiko
Woody shoot width	intermediate	small	large
Woody shoot colour	yellow or yellowish brown	dark brown	reddish brown
Bud fruitfulness after the 6th node	no	no	yes
Trend for suckers near the trunk base	no	yes	no
Shoot attitude	erect or drooping	semi-erect or semi-drooping	erect
Nonoccurrence of buds at nodes	no	no	some types
Trichomes on leaf surface	present in some types	no	yes
Shape of blade	wedge-shaped, pentagonal	pentagonal or reniform	pentagonal or circular
Shape of teeth	rectilinear or one side concave, one side convex	both sides concave or both sides convex or mixture	one side convex or mixture
General shape of petiole sinus	Closed or Lobes half overlapping	Very wide open or Wide open or Lobes slightly overlapping	Closed
Inflorescence	male and female fully developed	male and female fully developed	female with straight stamens or female with reflexed stamens
Berry shape	Elliptic or Oblate or Ovate	Obtuse-ovate or Obovate	Round or Oblate
Berry colour	green-yellow	blue black	blue black

Leaf diversity across accessions

Xynisteri

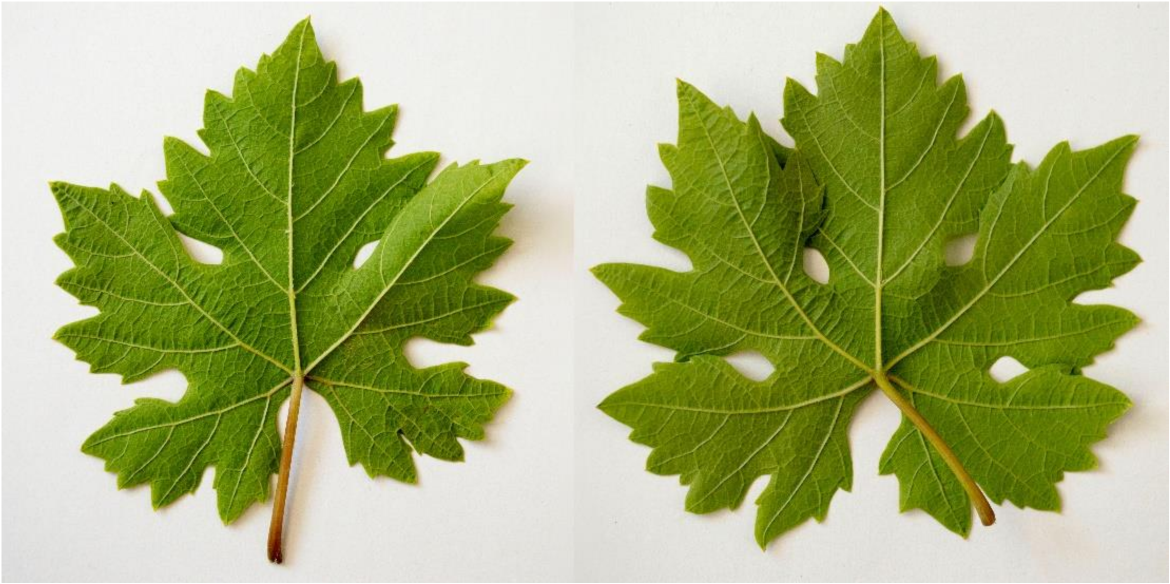

Mavro

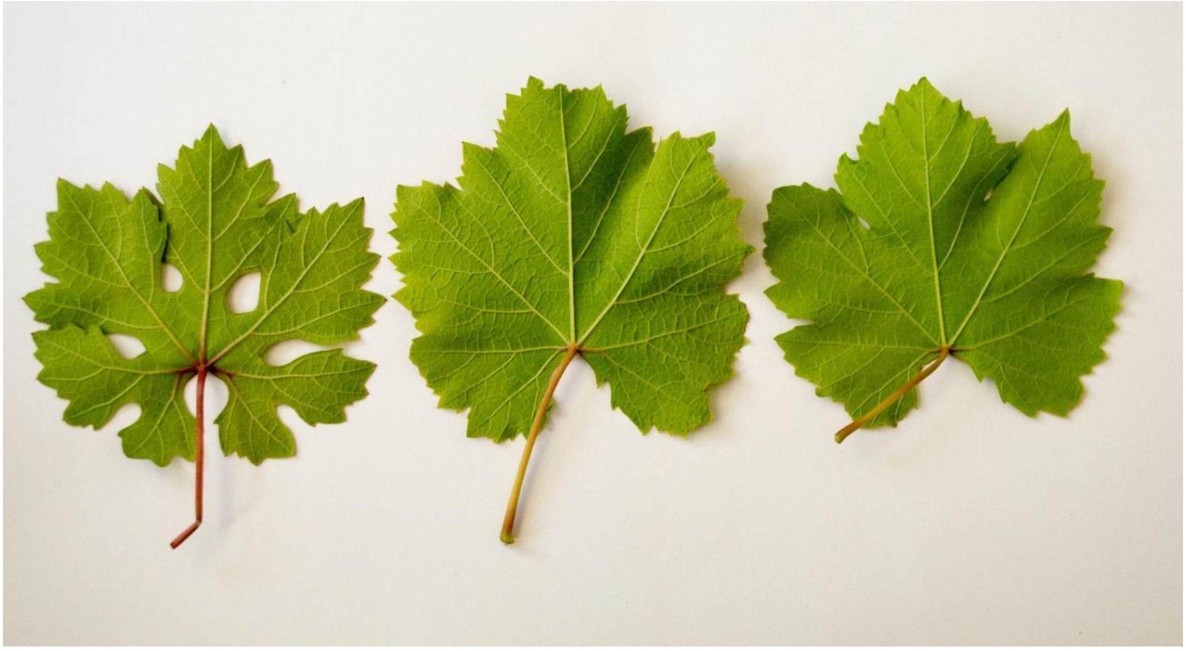

Maratheftiko

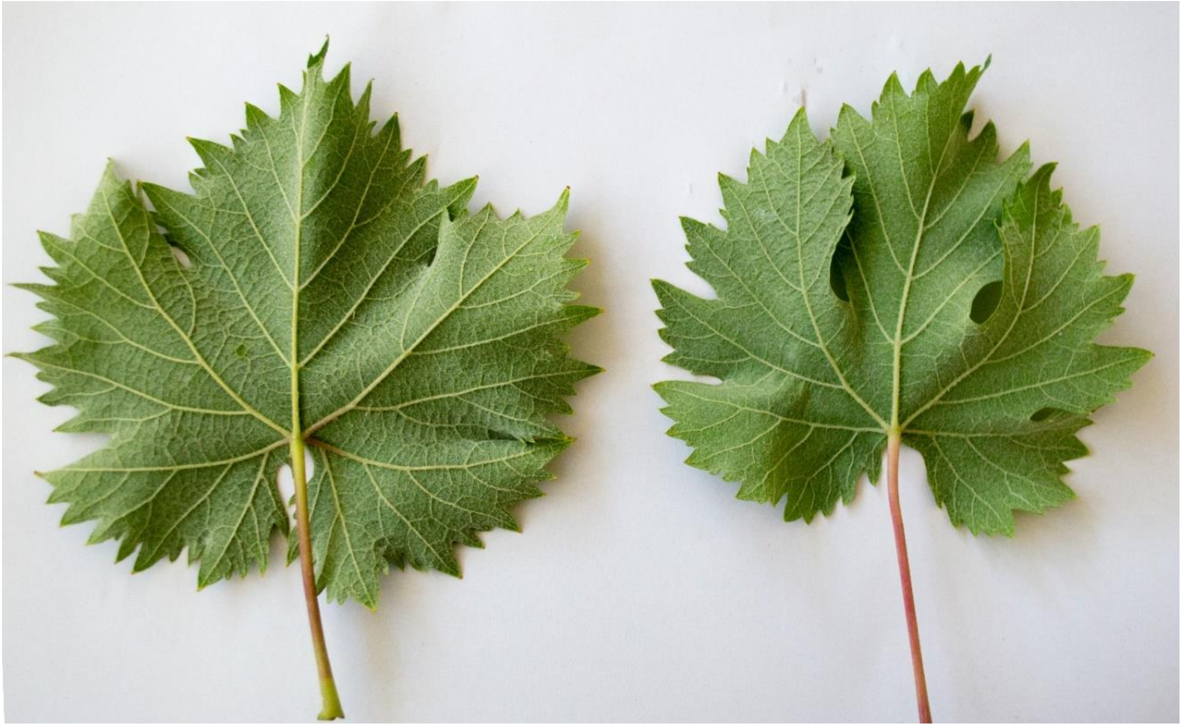

Supplement: Supplementary file 1 [file plants-09-01034-s001.zip › plants-821124 - supplementary data for XML.pdf]
